# U.S. POINTER: Topline Results of a Large Multisite Randomized Controlled Multidomain Lifestyle Intervention Trial

**DOI:** 10.1002/alz70860_096524

**Published:** 2025-12-23

**Authors:** Mark A. Espeland, Heather M Snyder, Laura D Baker, Rachel A. Whitmer, Miia Kivipelto, Ashley Alexander, Susan Antkowiak, Maryjo Cleveland, Claire E Day, Richard Elbein, Sarah Tomaszewski Farias, Deborah M Felton, Darren R. Gitelman, Marjorie Howard, Katherine Lambert, Laura Lovato, Xiaoyan Iris Leng, Olivia Matongo, Ann Marie McDonald, Kathryn V Papp, Valory Pavlik, Rema Raman, Stephen Salloway, Christy C Tangney, Benjamin J. Williams, Rena R. Wing, Robert A. Rissman, Sharon Wilmoth, Nancy Woolard, Melissa Yu, Maria C. Carrillo, the U.S. POINTER Study Group

**Affiliations:** ^1^ Wake Forest School of Medicine, Winston‐Salem, NC, USA; ^2^ Alzheimer's Association, Chicago, IL, USA; ^3^ Wake Forest University School of Medicine, Winston‐Salem, NC, USA; ^4^ University of California, Davis, Davis, CA, USA; ^5^ Karolinska Institutet, Stockholm, Sweden; ^6^ Kelsey Research Foundation, Houston, TX, USA; ^7^ Alzheimer's Association, Watertown, MA, USA; ^8^ Alzheimer's Association, San Jose, CA, USA; ^9^ Alzheimer's Association, Houston, TX, USA; ^10^ University of California, Davis, Sacramento, CA, USA; ^11^ Wake Forest University School of Medicine, Winston Salem, NC, USA; ^12^ Advocate Health, Downers Grove, IL, USA; ^13^ Alzheimer's Association, Western Carolina Chapter, Charlotte, NC, USA; ^14^ Alzheimer's Association, Illinois Chapter, Chicago, IL, USA; ^15^ Alzheimer's Association Houston & Southeast Texas Chapter, Houston, TX, USA; ^16^ Brigham and Women's Hospital, Boston, MA, USA; ^17^ Massachusetts General Hospital, Harvard Medical School, Boston, MA, USA; ^18^ Baylor College of Medicine, Houston, TX, USA; ^19^ Alzheimer's Therapeutic Research Institute, University of Southern California, San Diego, CA, USA; ^20^ Brown University, Providence, RI, USA; ^21^ Butler Hospital, Providence, RI, USA; ^22^ Rush University, Chicago, IL, USA; ^23^ Miriam Hospital, Providence, RI, USA

## Abstract

**Background:**

The U.S. Study to Protect Brain Health through Lifestyle Intervention to Reduce Risk (U.S. POINTER) is a Phase 3, multicenter, 2‐year, randomized controlled trial assessing the effects of two multidomain lifestyle interventions on cognition in older adults at risk for cognitive decline due to well‐established factors such as sedentary behavior, suboptimal diet, and suboptimal cardiometabolic health. A key aim was to test the generalizability of the FINGER findings in a diverse, representative U.S. cohort. Interventions were modeled on FINGER, adapted to U.S. culture, and delivered in collaboration with community partners.

**Method:**

Participants aged 60‐79 years meeting pre‐specified risk criteria for cognitive decline were randomized (1:1) at five sites to one of two intervention arms. Both arms targeted physical activity, nutrition, cognitive/social challenge and cardiometabolic risk management, but differed in intensity and accountability. The Structured arm (STR) included frequent peer team meetings, regular exercise, the MIND diet, BrainHQ cognitive training, social/cognitive challenge, and monthly blood pressure monitoring. The Self‐Guided arm (SG) included fewer peer team meetings and general health education with support to encourage health behaviors. The primary outcome, assessed at baseline and every six months, was change in global cognition, measured using a composite score based on tests of executive function, episodic memory, and processing speed. Secondary outcomes included domain‐specific cognitive scores, Clinical Dementia Rating‐Sum of Boxes, functional ability, frailty, and intervention response by subgroups (e.g., APOE4 carriers).

**Result:**

A total of 2,111 participants (mean age 68±5 years, 69% female, 31% from minoritized ethnoracial groups) were randomized. Retention exceeded 94%, and intervention adherence remained high throughout the 2‐year intervention period. Topline findings on intervention effects for primary and secondary outcomes will be presented. Additionally, two participants will share testimonials about their trial experiences.

**Conclusion:**

U.S. POINTER, the largest lifestyle intervention trial globally, successfully met ambitious recruitment goals and engaged a diverse, at‐risk cohort while maintaining high retention and participation rates. The topline results will provide pivotal evidence on the effectiveness of multidomain interventions to improve cognition or slow decline in older U.S. adults, with high potential to inform public health initiatives aimed at reducing Alzheimer's disease and related dementia risk.

